# Peer Review of “The Performance of DeepSeek R1 and Gemini 3 in Complex Medical Scenarios: Comparative Study”

**DOI:** 10.2196/96227

**Published:** 2026-04-27

**Authors:** Mayank Kejriwal

**Affiliations:** 1 University of Southern California, Los Angeles, CA, United States

**Keywords:** large reasoning model, LRM, large language model, LLM, accuracy, medical scenario, DeepSeek R1, Gemini 3

*This is a peer-review report for “The Performance of DeepSeek R1 and Gemini 3 in Complex Medical Scenarios: Comparative Study.”*

## Round 1 Review

### General Comments

This paper [1] reports on an experimental study to analyze the Massive Multitask Language Understanding Pro (MMLU-Pro) Q&A dataset. The authors find that DeepSeek R1 had an accuracy rate of 95.1% in 162 medical scenarios after reconciliation with subject matter experts on 23 questions. The findings contribute to the growing body of knowledge on large language model applications in health care and provide insights into the strengths and limitations of DeepSeek R1 in this domain.

### Specific Comments

#### Major Comments

1. The results are not appropriately qualified with results on statistical significance, and/or are lacking comparisons with other language models. Even if we know how other models perform overall, it would still be good to have more details, such as a comparison of where one model is right and another is wrong. Those kinds of deep insights are lacking in this paper. All we really know is that DeepSeek performs at a level roughly equivalent to the other leading models (nothing surprising there) and that it sometimes has incomplete or inexplicable behavior. I feel the paper needs to have more results and analysis to be a good fit for this journal.

2. Maybe you could add a workflow diagram/figure to better illustrate the methods?

3. I would like Table 1 to be augmented. Perhaps you can add an example question with answer choices? Right now, it looks very trivial. The alternative is to create a simple bar graph instead of a table, but the former would be more useful.

## Abbreviations

MMLU-Pro: Massive Multitask Language Understanding Pro
